# Factors That Affect the Acceptance of Educational AI Tools by Greek Teachers—A Structural Equation Modelling Study

**DOI:** 10.3390/ejihpe14090169

**Published:** 2024-09-21

**Authors:** Katerina Velli, Kostas Zafiropoulos

**Affiliations:** Department of Educational & Social Policy, University of Macedonia, 546 36 Thessaloniki, Greece; katvel2109@gmail.com

**Keywords:** educational AI tools (EAIT), teachers, perceived usefulness, perceived ease of use, perceived trust, personal innovativeness, social influence, facilitating conditions, pedagogical beliefs, PLS SEM, Greece

## Abstract

The discussion around integrating AI technologies into educational practice is current among scholars and in sociopolitical circles. This study examines the factors influencing teachers’ acceptance of educational AI tool (EAIT) use, aiming to inform the development of a pedagogical framework for the responsible integration of AI tools in education. A conceptual model was developed by amalgamating constructs of TAM (perceived usefulness and perceived ease of use) and UTAUT (social influence and facilitating conditions) while integrating the variables of perceived trust and personal innovativeness and considering the impact of teachers’ pedagogical beliefs. A total of 342 Greek teachers participated in the quantitative survey conducted. The proposed model was evaluated using partial least squares structural equation modelling (PLS-SEM). The findings illuminated perceived usefulness as the most significant predictor of teachers’ behavioural intention to use EAIT. The research also revealed that social influence and personal innovativeness exert considerable influence. While constructivist pedagogical beliefs were found to have no direct impact on EAIT acceptance, the results indicated that educators who embrace those teaching methods exhibit a high propensity to perceive EAIT as useful and trustworthy. Furthermore, the study’s analysis demonstrated that trust had a significantly positive effect on usefulness, and innovativeness influences positively and significantly both usefulness and ease of use.

## 1. Introduction

The field of artificial intelligence (AI) has recently experienced a period of rapid advancement driven by the convergence of three key factors: the availability of big data, the advent of affordable computing power, and the emergence of groundbreaking advances in machine learning [1]. The surge in AI capabilities has led to its integration into various aspects of our lives, including facial recognition technology and self-driving cars [2]. It is important to note that the proliferation of AI is not merely a technological phenomenon; it is a societal shift that is reshaping industry, the economy, and daily routines [3].

In the context of global employability trends and the impact of digital transformation, it is anticipated that AI will be embraced by the majority of enterprises at a rate of 75% within the near future. This will result in significant transformations, either through the emergence of novel positions or the substitution of existing ones [4]. The transition of numerous employees to upskilled professional categories requires the development of entrepreneurial competencies, the establishment of a legal infrastructure, the enhancement of open innovation, and global collaboration [3]. Technological upheaval will result in a multitude of ongoing modifications to lifestyles, societal institutions, norms, curriculum, and educational modalities [5,6].

Similarly, the field of education is not immune to AI’s transformative influence. The integration of AI into education, particularly through intelligent tutoring systems (ITS), has gained considerable momentum. Initially, these systems were teacher-centred, but they have since evolved to become student-centric, adapting to individual learning styles and needs [7]. This has paved the way for the development of state-of-the-art AI tools.

AI systems are defined by two key features: autonomy and adaptability. Autonomy refers to the capacity to perform complex tasks without the need for constant guidance, while adaptability encompasses the ability to enhance performance through the acquisition of experience [8]. Educational AI tools (EAIT) can be broadly classified into three categories: learner-facing, teacher-facing, and system-facing. Learner-facing tools, such as personalised learning platforms and adaptive learning systems, directly support students by tailoring content and providing feedback. Teacher-facing tools, like automated grading systems and content recommendation engines, assist educators in their instructional practices. System-facing tools, including data analytics platforms and timetable generation software, help educational institutions manage and organise administrative tasks [9].

The potential of EAIT offers the prospect of alleviating teachers’ workload, thereby enabling them to concentrate on innovative teaching methods and differentiated instruction, with the intention of leveraging student engagement [9,10]. Therefore, the utilization of AI systems should be oriented towards pedagogical objectives rather than being limited to technological possibilities [11]. The integration of AI-powered tools into student performance enables educators to implement adaptive learning techniques, thereby fostering a dynamic and engaging learning environment. While AI streamlines mundane tasks and enhances efficiency, the irreplaceable role of teachers as supporters and facilitators remains essential for the holistic development of learners [12]. This synergy between EAIT and educators ensures that technology serves to enhance rather than supplant the human element in education, ultimately leading to a more effective and fulfilling educational experience for all [13].

The advent of ChatGPT, the most renowned example of a generative AI tool, has attracted significant attention from the educational community. Released to the public by OpenAI in November 2022, ChatGPT represents a large language model capable of generating text that is humanlike in its style, based on a given prompt [14]. Although it offers a multitude of applications ranging from content creation and assessment to language learning and personalised tutoring, its increasing use by learners has given rise to debates on academic integrity, privacy, and plagiarism concerns, as well as over-dependence issues [9,15]. This has led to discussions about the need for educators to adapt their assessment practices to ensure fair and accurate evaluation of student learning [16]. Additionally, the perceived lack of transparency in how EAIT operate and their misalignment with some curriculum objectives can create barriers to their adoption [17]. The “black-box” nature of the majority of AI algorithms, particularly those based on deep learning, presents a significant obstacle for educators attempting to comprehend the decision-making processes of these tools and to develop effective methods of integrating them into pedagogical practices [2]. The potential biased outcomes and ambiguity in effective access may impede teachers’ trust in EAIT and their willingness to incorporate them into their classrooms [18].

In order to harness the benefits of AI in education in a responsible manner, it is of utmost importance to establish robust ethical guidelines. Recently, there have been initiatives among a number of countries (Australia, China, Estonia, South Korea, and the U.S.A.) for the development of national strategies for AI integration in education, with the European Union being at the forefront of this effort, enacting thorough regulatory frameworks [1]. To facilitate the real-world development and implementation of educational AI, it is essential to foster collaboration among educational institutions, AI companies, and researchers. Furthermore, educators must be provided with comprehensive training and support in order to empower themselves to effectively utilise EAIT and leverage the potential to enhance teaching and learning [9]. By incorporating teachers’ expertise and responding to their concerns, EAIT frameworks can be developed to address the specific needs and pedagogical goals [19].

In the case of Greece, the educational system is centralised, with the Ministry of Education overseeing curriculum, teacher training, and assessment. Primary and secondary education is compulsory, emphasizing theoretical knowledge and examinations [20]. In recent years, the integration of technology in education has become a growing priority, yet significant challenges persist, particularly concerning infrastructure and teacher training. Despite government investments in technological resources, there remains a notable gap in teachers’ familiarity and comfort with these tools. Many educators recognise the potential benefits of technology in enhancing student engagement and learning outcomes, but without comprehensive training and support, the effective integration of technology into teaching practices is hindered. As Greece continues to navigate these challenges, fostering a supportive environment for both teachers and students is essential for realizing the full potential of technology in education [21,22].

The acceptance of technological innovation by users, especially within the domain of education, is of vital importance. The increasing prevalence of EAIT in educational settings has sparked growing interest among researchers and policy makers in understanding the factors that influence teachers’ acceptance and adoption of these tools [23]. The technology acceptance model (TAM) and the unified theory of acceptance and Use of Technology (UTAUT) are commonly employed theories in this area [24,25,26,27,28]. TAM emphasizes the significance of perceived usefulness and perceived ease of use [29], whereas UTAUT offers a more comprehensive framework by evaluating performance expectancy, effort expectancy, social influence, and facilitating conditions [30]. A common practice in quantitative surveys conducted on the basis of TAM and UTAUT is to extend the models in order to improve the interpretation of the relationships among the investigated factors [31,32]. The academic literature and previous research indicate that further factors are necessary for a thorough understanding of technology acceptance. The role of perceived trust in online technology, particularly concerning privacy and security in managing personal data [18,24,32,33], the effect of pedagogical beliefs encompassing educators’ values and attitudes towards teaching and learning [24,34,35,36,37,38], and the influence of personal innovativeness denoting the propensity to experiment with novice technologies ahead of others [39,40,41,42,43] can enrich the shaping of teachers’ attitudes towards AI.

Educational AI represents a novel and expanding field of research with identified gaps that warrant further exploration. International studies [25,27,28,31] have employed technology acceptance theories, both independently and extensively, to analyse patterns of AI adoption. Nevertheless, no research has hitherto combined variables from the traditional TAM and UTAUT models with trustworthiness, pedagogical approaches, and personal innovativeness. Moreover, the majority of field studies have focused on the tertiary education sector [28,44,45], with limited research investigating school education. Within the Greek educational context, research on AI remains scarce due to the early developmental stage of the technology. Nonetheless, in light of the transformative digital policy agenda in Greek services [46], the emergence of AI applications in pedagogical practices necessitates a systematic inquiry into school education.

The present study aims to address this gap through a quantitative research approach. The research focuses on Greek school teachers’ perspectives on AI technologies, and it selectively merges constructs from TAM, UTAUT, and the aforementioned supplementary factors. The aim is to advance our understanding of educators’ intentions to accept AI and facilitate the successful integration of EAIT in educational settings.

We propose a unified research model through the amalgamation of particular dimensions of two prior systematic reviews; the one conducted by Choi, Jang, and Kim [24] and the other by Strzelecki [42]. The conceptual framework draws on the components of the technology acceptance model (TAM), perceived usefulness (PU) and perceived ease of use (PEU), and incorporates two constructs from unified theory of acceptance and use of technology (UTAUT), social influence (SI), and facilitating conditions (FC). It is further augmented with some external variables, namely, perceived trust (PT), pedagogical beliefs (PB), and personal innovativeness (PI). Behavioural intention (BI) refers to the degree to which teachers intend to use EAIT in the teaching practice. For facilitating the reading of the paper, the acronyms of the variables are defined in Table 1. Details on the proposed model construction and hypotheses building follow.

### Hypotheses Formulation

TAM elucidates the concept of behavioural intention (BI) in the context of technology adoption, delineating the role of PU and PEU in this process. In the educational domain, PU pertains to the perceived utility of a tool in enhancing teaching performance, whereas PEU encapsulates teachers’ anticipation of the effortlessness associated with the tools’ utilisation [29]. A TAM-based inquiry on predicting teachers’ attitudes towards chatbot use yielded valuable findings, revealing a significant effect of both constructs PU and PEU [25]. Other research [26] highlighted the predictive strength of PU in investigating K-12 teachers’ readiness to introduce AI instruction. Most researchers integrate and extend TAM with other models or factors [47,48]. In particular, Prasetyo et al. [49] demonstrated the statistically significant effect of PEU on e-learning acceptance by high school students during the COVID-19 pandemic. Similarly, Choi et al. [24] revealed a significant influence of PEU on school teachers’ EAIT adoption. In the context of this study, we assess the impact of PU and PEU on Greek school teachers’ BI to use EAIT. The hypotheses developed are as follows:

**H1:**  *Perceived usefulness has a significant positive effect on behavioural intention*.

**H2:**  *Perceived ease of use has a significant positive effect on behavioural intention*.

The UTAUT model identifies four key factors for predicting the intention to use technology: performance expectancy (PE), effort expectancy (EE), social influence (SI), and facilitating conditions (FC). PE, corresponding to PU, is defined as the degree to which an individual using a technology believes it will benefit their work. EE, corresponding to PEU, refers to the level of ease associated with using the system. SI concerns the degree of influence that important others (the social environment) exert on the individual to use a technology. FC refer to the degree to which the individual believes that organisational and technological infrastructures support the use of a system [30]. Although the maturity level of TAM is demonstrably higher than that of UTAUT, the latter has been widely applied in information systems research, including those in educational settings [50]. The significance of PE, EE, and FC in driving the acceptance and use of technology in education has been consistently demonstrated by researchers, as evidenced in studies on mobilelearning [51,52], AI-assisted learning environments [41], and educational platforms like MS Teams [53]. However, the impact of SI on technology adoption appears less consistent, with some studies indicating its significance [45], while others find it negligible [51].

In his study, Strzelecki applied the extended UTAUT2 model and identified habit, PE, and hedonic motivation as key predictors of the adoption of ChatGPT by university students in Poland [42]. Given the characteristics of cutting-edge technology and the way in which users interact with EAIT, we have chosen to investigate the effect of SI and FC on teachers’ BI to use EAIT. When teachers receive adequate support and EAIT are readily accessible, FC are supported. In instances where teachers receive encouragement from colleagues, family, and friends, SI is perceived as a predictor of EAIT adoption. The factors of hedonic motivation and habit, as utilised in Strzelecki’s research, were not included in the present research due to the perceived irrelevance to the specific population and research field purpose. Teachers are more likely to adopt a technology based on its perceived usefulness and ease of use rather than on their personal enjoyment. Furthermore, the concept of habit is not applicable in the case of EAIT, as their use in education is relatively new and has not yet been established as an everyday practice. In this study, we preferred to include PU and PEU, i.e., the TAM constructs, rather than their conceptually overlapping PE and EE of the UTAUT model. This decision was made with the aim of improving the clarity and interpretability of the questionnaire items for the participating teachers. In our view, the terminology of the TAM constructs would be more intuitive and straightforward for investigating Greek teachers’ attitudes towards the use of a state-of-the-art technology as EAIT, and therefore, more accurate and meaningful conclusions would be elicited. Consequently, the hypotheses developed are as follows:

**H3:**  *Facilitating conditions have a significant positive effect on behavioural intention*.

**H4:**  *Social influence has a significant positive effect on behavioural intention*.

The concept of perceived trust (PT), defined as the level of reliability users have in a system, is a crucial factor in technology acceptance, particularly in the online environment where personal data and privacy security are paramount [54]. In the field of educational AI, PT is vital to test due to the ethical challenges associated with using AI tools [33]. To mitigate algorithmic opacity and harness the full potential of EAIT, it is essential to educate teachers and establish regulations for AI systems [55]. Nazaretsky et al. [18] explored teachers’ trust in an AI-supported blended teaching model and determined that obscurity of AI decisions is a limiting factor, while adjusting teachers’ pedagogical methods can increase trust. Researchers [24,48] have also attempted to merge trust into the TAM structure. For instance, Sánchez-Prieto et al. [48] developed a theoretical TAM-based model expanded with trust, among other external factors, to investigate learners’ views on their acceptance of EAIT as an assessment method. Similarly, the study conducted by Choi et al. [24] revealed a significant impact of teachers’ PT on PU and BI to use EAIT, while receiving a positive influence from PEU. In the light of the existing literature, it is evident that there is a need to investigate the influence of PT in the survey of teachers’ acceptance of EAIT. It can be reasonably assumed that teachers’ confidence in the reliability of EAIT affects their adoption decisions, while lack of confidence may lead to reluctance or rejection despite the benefits of EAIT. Therefore, the following hypotheses are proposed:

**H5:**  *Perceived trust has a significant positive effect on perceived usefulness*.

**H6:**  *Perceived trust has a significant positive effect on behavioural intention*.

Teachers’ pedagogical beliefs (PB), significantly influenced by their knowledge (of content and instruction) and professional experience, shape their educational practices, their decision-making processes, and their interactions with learners [56]. In the field of educational technology, they are broadly categorised as traditional pedagogical beliefs (TPB), namely, teacher-centred approaches, and constructivist pedagogical beliefs (CPB), that is, student-oriented ones. TPB emphasise the role of teachers in guiding knowledge acquisition through reinforcement, whereas CPB are rooted in the active participation and cooperation of the learners in instructional activities [57]. The literature denotes that teachers with constructivist approaches are more likely to adopt innovative technologies, such as AI, in their classroom, and in fact they tend to use them in a more creative and student-centred way [34,35].

The integration of PB into TAM is regarded as a valuable approach to optimise our comprehension of teachers’ perceptions towards any technological system [36]. Recent studies have revealed a stronger consensus among teachers in favour of CPB compared to TPB. Furthermore, it was demonstrated that CPB can positively influence PU, PEU, attitude, and BI to use ICT or EAIT, whereas TPB exhibited either a negative or a minimal positive impact [24,37,38]. In order to measure the tendency to adopt EAIT, the external variables of teachers’ CPB and TPB were drawn into the framework of our educational AI research. When teachers’ PB are aligned with the potential of EAIT, it is more possible to integrate the tools into their tasks. As a consequence, the formulation of hypotheses is as follows:

**H7:**  *Constructivist pedagogical beliefs have a significant positive effect on perceived usefulness*.

**H8:**  *Constructivist pedagogical beliefs have a significant positive effect on perceived ease of use*.

**H9:**  *Constructivist pedagogical beliefs have a significant positive effect on perceived trust*.

**H10:**  *Constructivist pedagogical beliefs have a significant positive effect on behavioural intention*.

**H11:**  *Traditional pedagogical beliefs have a significant negative effect on perceived usefulness*.

**H12:**  *Traditional pedagogical beliefs have a significant negative effect on perceived ease of use*.

**H13:**  *Traditional pedagogical beliefs have a significant negative effect on perceived trust*.

**H14:**  *Traditional pedagogical beliefs have a significant negative effect on behavioural intention*.

Empirical studies have demonstrated that teachers’ willingness to adapt and experiment can motivate them to adopt pioneering instructional approaches, including the acceptance of information technology innovations, such as e-learning and ChatGPT [40,42]. Although TAM and UTAUT are fundamental to technology acceptance research, they frequently fail to consider the impact of personal innovativeness (PI), which is rooted in the diffusion of innovation theory (DOI) and refers to early adopting and disseminating any technological advancement [58]. Given the avant-garde nature of EAIT, incorporating PI into models for predicting teachers’ acceptance is crucial for interpreting their adoption behaviours [39]. Therefore, we suggest the following hypotheses:

**H15:**  *Personal innovativeness has a significant positive effect on perceived usefulness*.

**H16:**  *Personal innovativeness has a significant positive effect on perceived ease of use*.

**H17:**  *Personal innovativeness has a significant positive effect on behavioural intention*.

## 2. Materials and Methods

**Figure 1:**
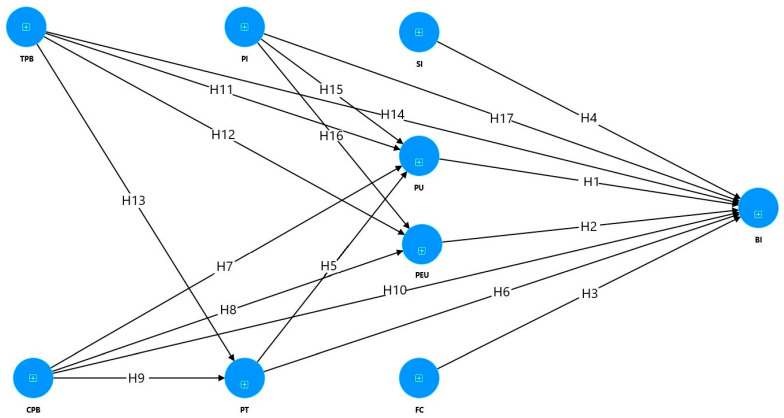
The proposed research model.

The methodology employed in this research is deductive, entailing the formulation of hypotheses at the outset followed by empirical investigation using data. Our conceptual model and the hypotheses are presented in Figure 1.

### 2.1. Participants and Sample Descriptives

The participant group of the study consisted of teachers serving at primary and secondary schools under the supervision of the Greek Ministry of Education. The research focused on investigating the demographic factors of gender, age, academic qualifications, teaching experience, and participation in training programmes on AI technology (degree studies, micro-masters, MOOCs, webinars, etc.). The rationale for excluding certain other profile information, such as education level (primary, secondary, or vocational), education domain (literature, science, mathematics, foreign languages, etc.), employment status (permanent or temporary position), and school location, lies in the following considerations: while the excluded factors might influence familiarity with technology (particularly in relation to education level and domain) or possibly correlate with access to resources and potential professional development opportunities (in relation to school location and employment status), the study argues that its primary goal is to understand individual-level perspectives and attitudes, and focus on broader acceptance patterns, which may transcend any differences in job position or subject expertise.

A total of 342 teachers responded in the survey. A gender distribution analysis of the participants revealed that the majority were female (77.5%), reflecting the numerical superiority of women teachers in Greece over men, with one participant preferring to withhold their gender. With regard to age, 41.5% of participants were over 51 years old, one third were 41–50 years old, and the remainder were below the age of 40. Referring to academic qualifications, half of the sample had completed postgraduate studies; roughly one in ten had earned a PhD or were PhD candidates (9.4%), while the remaining 40.6% had gained a bachelor’s degree. Based on the teaching experience, approximately equal percentages were in the 21–30 years (32.5%) and 11–20 years (29.5%) groups. Roughly, one-quarter of the teachers had less than 10 years of experience (24.3%), while 13.7% had more than 31 years of teaching experience. Finally, at the time of the survey, one-third of the sample (33.6%) had participated in a typical or non-typical professional development programme (PD) on AI expertise, whether completed or still in progress. This may be due to the recent evolution of AI tools regardless of the initiatives on teacher professional development held by universities and any other public or private educational institutions (Table 2).

### 2.2. Instrument of the Study

A quantitative empirical study was conducted using an online questionnaire. The convenience sampling method was employed. The questionnaire was distributed to school email lists, requesting each school principal or mailing manager to forward the link to teachers’ personal emails. In addition, the link was shared within teachers’ groups on social networks and teachers’ learning online communities. The study instrument was accompanied by an introductory consent form, which informed the research subjects about the study purpose, their voluntary and anonymous participation, their personal data protection, and the absence of any risk, cost, or reward. In order to verify that the research project content did not contravene the current legislation in terms of ethical principles, the researchers submitted an application to the university’s research ethics committee (REC), which issued the petition approval (No. 22/17.1.2024). The questionnaire was designed to take approximately 10 min to complete. Data collection commenced on 18 January 2024 and concluded on 29 February 2024.

The study instrument was constructed from the research instruments of two previous related studies [24,42]. It was translated into the Greek language for the convenience of the participants, modified, and adapted as appropriate to align with the context of the particular field study. It consists of 9 sections, which include 36 items measuring teachers’ perceptions on TPB, CPB, PI, PT, PU, PEU, SI, FC, and BI. Totally 25 items were adopted from Choi et al. study [24], while 11 items were taken from Strzelecki study [42]. In the latter, the term “ChatGPT” was replaced with the acronym “EAIT”. The measurement scales (Table 3) were rated on a 5-point Likert scale from 1 indicating “strongly disagree” to 5 indicating “strongly agree”.

### 2.3. Statistical Analysis

The study team employed structural equation modelling, analysed with SmartPLS software application v. 4.1.0.6 [59] to evaluate the measurement model, first in terms of its reliability and validity, and then in order to verify the proposed assumptions. Some sources posit that the minimum sample size for SEM analyses is 10 times the number of model items, while others suggest that the sample size should be at least 200 [60]. In fact, the greater the complexity of the model, the larger the sample size should be [61]. In the context of the present study, the sample size of 342 observations is considered satisfactory, given that our model comprises 36 indicators.

### 2.4. Reliability and Validity of the Measurement Model

Cronbach’s alpha, rho A, and composite reliability coefficients are used to assess the reliability of scales [62]. Each component in Table 4 exhibits internal consistency. All values are above the recommended threshold of 0.7, denoting good reliability. Each of the constructs has sufficient convergent validity, as indicated by the average variance extracted (AVE) values, which are all greater than the threshold of 0.5.

The Fornell–Larcker criterion, the cross-loadings criterion, and the heterotrait-monotraitratio of correlations (HTMT) criterion are used to examine discriminant validity. For the Fornell–Larcker criterion, the correlation matrix of all constructs is calculated, and for each concept, the correlations must be less than the square root of the average variance extracted (AVE). The cross-loadings criterion analyses the loadings of the indicators that represent a construct. Each construct should have higher loadings than the indicators that reflect the concept it represents. The HTMT criterion computes the mean correlations between indicators measuring the same construct, compared to the mean correlations between indicators across different constructs. Values should be less than 0.85 to confirm discriminant validity [62].

Table 5 presents the results regarding the Fornell–Larcker criterion. The condition is met, as all correlations are less than the square roots of the average variance extracted (AVE, written in bold) of the relevant constructs. The cross-loadings, as shown in Table 6, provide evidence of discriminant and convergent validity. Each concept shows strong loadings only on the elements it is supposed to represent. Table 7 shows the HTMT values, all of which are less than 0.85. Overall, in our analysis, discriminant validity is confirmed regarding all the aforementioned criteria.

## 3. Results

### 3.1. Structural Model Analysis

The structural equation model (SEM) analysis utilises SmartPLS software as a robust statistical tool, particularly for investigating complex relationships between variables, even within small sample sizes and non-normal data distribution. This is possible due to the iterative optimization process for the PLS-SEM algorithm, focusing on gradual prediction rather than model fit [63]. The procedure entails an examination of the model’s both explanatory and predictive power. The coefficient of determination R^2^ measures the model’s explanatory power, with values ranging from 0 to 1; a higher value indicates greater explanatory power. Exceptionally high R^2^ values (around 0.90) are not normally expected in models that predict attitudes and intentions of a population [62], as observed in the present research model. A non-parametric resampling procedure, recommended by Hair and Alamer [63], does not require data normality and is known as the bootstrapping method. It was performed to assess the statistical significance of the PLS-SEM results and to estimate the path coefficients. This routine was carried out using 5000 sampling repetitions.

**Figure 2:**
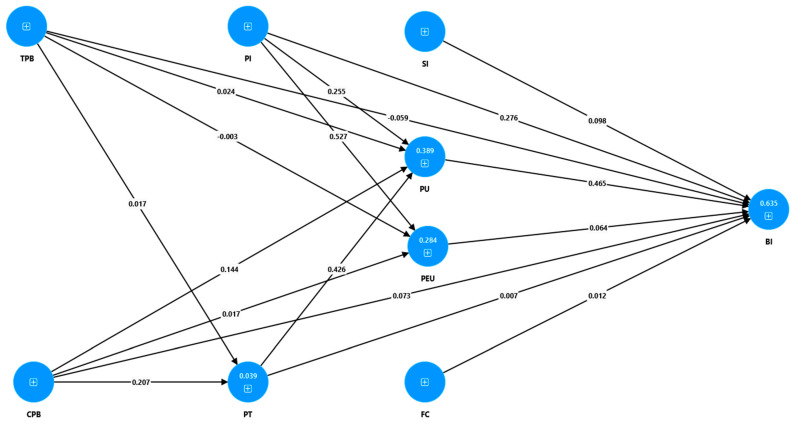
Path coefficients of the research model and direct effects results.

The coefficient of determination (R^2^) is assessed to quantify the variance in outcomes explained by predictors, considering the model constructs’ interpretation and potential overfitting concerns. R^2^ values are contextdependent and interpreted with suggested thresholds of 0–0.10, 0.11–0.30, 0.30–0.50, and >0.50 from weak to strong explanatory power [63]. As illustrated in Table 8 and Figure 2, the highest R^2^ value was observed for the behavioural intention (BI) (R^2^ = 0.635, *p* = 0.000), indicating that the variables included, and mostly perceived usefulness (PU, *β* = 0.465) and personal innovativeness (PI, *β* = 0.276), account for a significant 63.5% of the variance in BI. The remaining endogenous variables exhibited R^2^ values that vary from modest to low, yet retained statistical significance. Notably, the model demonstrated moderate and modest R^2^ for the perceived usefulness (PU) and perceived ease of use (PEU) endogenous variables (R^2^ = 0.389 and R^2^ = 0.284, respectively). The R^2^ for the model with the PT as the dependent variable (R^2^ = 0.039) was not statistically significant.

We also assessed collinearity among variables using the VIF index, which indicates the degree to which these variables correlate with each other. According to Table 9, we found that all VIF values were below the threshold of 5, which means that all levels of collinearity were acceptable.

### 3.2. Hypothesis Testing Results

Based on the hypotheses proposed in this study, the subsequent phase is the evaluation of the structural model. The direct, indirect, and total effects are set out in Table 10. The total effects of the predictor variables are between −0.045 and 0.527, comprising the sums of direct and indirect effects.

Of the seventeen hypotheses formulated, regarding the direct effects, eight were supported, while nine hypotheses were not supported. The following direct effects are positive and statistically significant: PU -> BI (*β* = 0.465, *p* = 0.000), SI -> BI (*β* = 0.098, *p* = 0.035), PT -> PU (*β* = 0.426, *p* = 0.000), supporting H1, H4, and H5, respectively. Furthermore, CPB has a positive and statistically significant effect on PU (*β* = 0.144, *p* = 0.013) and on PT (*β* = 0.207, *p* = 0.002). These findings support H7 and H9, respectively. In addition, the results indicate that PI influences positively and with statistical significance PU (*β* = 0.255, *p* = 0.000), PEU (*β* = 0.527, *p* = 0.000), as well as BI (*β* = 0.276, *p* = 0.000), verifying hypotheses H15, H16, and H17, respectively. Conversely, no statistically significant direct influences were observed of CPB on BI, CPB on PEU, FC on BI, PEU on BI, or PT on BI. This analysis leads to not supporting the hypotheses H10, H8, H3, H2, and H6, respectively. The results yielded no evidence that TPB exerts any influence on PU, PEU, PT, or BI, and thus H11–H14 were not supported, yet with a negative sign for the relations of TPB on BI and PEU.

Furthermore, the indirect and total effects of the variables were analysed. PU was identified as the most dominant determinant of teachers’ BI to use EAIT (*β* = 0.465, *p* = 0.000), followed by PI (*β* = 0.428, *p* = 0.000), PT (*β* = 0.205, *p* = 0.000), and CPB (*β* = 0.183, *p* = 0.001). Additionally, there were other total positive and statistically significant effects, including PI -> PEU (*β* = 0.527, *p* = 0.000), PT -> PU (*β* = 0.426, *p* = 0.000), PI -> PU (*β* = 0.255, *p* = 0.000), CPB -> PU (*β* = 0.232, *p* = 0.000), CPB -> PT (*β* = 0.207, *p* = 0.002), and SI -> BI (*β* = 0.098, *p* = 0.035).

## 4. Discussion

The purpose of this study was to delve into the factors that shape teachers’ acceptance of EAIT, focusing on the SEM analysis method using the SmartPLS tool. The conceptualization of the research model presented is unique in that it integrates TAM constructs (perceived usefulness and ease of use), specific UTAUT variables (social influence and facilitating conditions), and other critical factors, such as perceived trust, personal innovativeness, and pedagogical approaches. Data analysis of all nine variables revealed high construct reliability, while acceptable convergent and discriminant validity standards were also met. This gives the researchers confidence that the measurement instrument is a strong and accurate tool, ensuring that the results are consistent and can be used to draw meaningful conclusions about the factors influencing teachers’ acceptance of AI tools.

At first, we investigated hypotheses regarding the relationships between TAM constructs. The analysis revealed that perceived usefulness (PU) is the most influential factor in determining teachers’ behavioural intention (BI) to use EAIT, while perceived ease of use (PEU) has no significant relationship with the intention to use. This finding is partially consistent with TAM theory, which posits that both PU and PEU are primary determinants of technology acceptance [29]. Prior research on teachers’ use of AI in K-12 schools lends support to the assertion that PU is a significant factor, yet in a model that does not include the PEU variable as a predictor [26].When teachers perceive EAIT as beneficial and enhancing work performance, they are more likely to adopt and integrate these tools into their teaching practices, despite the fact that they do not necessarily consider the tools to be user-friendly. In practical terms, PEU is presumably not the most crucial factor for Greek teachers, especially those who are less technologically inclined. This assumption contradicts findings of previous studies, which indicated that PEU of EAIT is a significant determinant in their adoption [24,25]. It is also worth noting that in our research, PU plays a mediating role towards BI, exerting an indirect and total effect from perceived trust (PT) and personal innovativeness (PI). This evidence suggests that teachers, who tend to trust EAIT and feel innovative in their use, find EAIT useful, which in turn increases their intention to accept them. Besides PU having a direct effect on BI, it serves as a mediating variable. The indirect and total effects on BI from constructivist pedagogical beliefs though PU imply that teachers who implement student-centred approaches in the classroom are likely to consider EAIT effective, thereby promoting their widespread adoption.

Furthermore, the study highlights the role of personal innovativeness (PI) in teachers’ acceptance of EAIT. It is noteworthy that PI not only directly influences BI but also affects it indirectly via PEU and PU. Teachers who are more open to new experiences and willing to explore and experiment with novel technologies are more likely to perceive EAIT as beneficial and to find them easy to use. The multifaceted impact of PI is reflected in previous studies, which also implemented PI into TAM constructs [39,40,58]. These studies found that teachers’ openness to new technological experiences led to a positive evaluation of innovations, which in turn affected their intention to adopt them [39,41,58].

In order to enrich the empirical research, two additional UTAUT model factors, namely, social influence (SI) and facilitating conditions (FC), were explored with a view to triggering teachers’ acceptance of EAIT. With regard to SI, our analysis showed a positive and statistically significant effect on BI, suggesting that the positive opinions and encouragement of colleagues, family members, and friends can influence teachers’ decisions to adopt EAIT. This result is parallel to the observations of other researchers who affirmed the significant impact of students’ SI on their willingness to accept educational AI technology [45] and ChatGPT use [42].This highlights the importance of creating a supportive social environment for teachers who are considering the adoption of EAIT. As for the FC variable, the testing yielded no significant direct effect on BI. This outcome is in accordance with the findings of Strzelecki’s study [42]. In the context of the Greek educational setting, where the level of technological infrastructure and resources in schools is relatively basic, it is surprising that teachers do not perceive a significant difference in technical availability and compatibility for the potential use of EAIT. Moreover, the pedagogical advantage of an AI tool may be a more dominant factor in teachers’ views than the ease of access or lack thereof. Prior research reported that factors such as undisturbed access to resources and adequate technical support can influence technology adoption [42] and the acceptance of mobile learning among students [51].

An intriguing finding of the study was that perceived trust (PT) did not have a statistically significant direct effect on teachers’ BI to use EAIT. It is, however, important to note that the analysis revealed an indirect effect of PT on BI through PU. This shows that while PT may not directly influence teachers’ intention to use EAIT, it can directly affect their perception of the practical value of these tools. The result is partly in line with Choi et al.’s work [24], who pointed out the direct positive and significant effects of PT both on PU and BI. This illustrates that when teachers believe in the reliability and fairness of EAIT, they are more inclined to use them. Further research approaches have underlined the key role of trust in acceptance of educational AI. Some research has focused on the factors that influence trust per se in AI systems [18,33], while others have developed theoretical models [48] or built upon preliminary dataset results [32]. This review underscores the necessity for further studies on the impact of transparent and explainable AI algorithms in education, as well as the importance of addressing ethical concerns related to data privacy and algorithmic bias.

Complementary to the models proposed in the literature review, this study was selected to examine the direct effects of pedagogical beliefs (PB) on BI. Nonetheless, the results have been unable to demonstrate a significant direct influence of constructivist pedagogical beliefs (CPB), but rather a total and indirect one. In spite of the aforementioned, the CPB factor was noticed to play a key role in PU, exerting direct, indirect, and total effects on it, as well as a significant direct impact on PT but not any effect on PEU. Prior research works are partially aligned to our analysis results [24,37,38]. This discrepancy implies that educators who favour student-centred and inquiry-based pedagogical approaches are more likely to regard EAIT as useful and trustworthy, even though they are not guided by the perception of effortless tool operation.

Additionally, no significant negative influence of traditional pedagogical beliefs (TPB) was observed on teachers’ predisposition to use EAIT or on any other variable, contrary to conclusions of earlier studies [24,37,38]. Since this outcome has not been found in other studies, it could be attributed to the evolving nature of pedagogical approaches, with many teachers adopting a blended teaching style that combines elements of both traditional and constructivist pedagogies. It is, therefore, proposed that professional development programmes that promote CPB should be developed in order to facilitate the integration of EAIT into educational settings.

### 4.1. Limitations of Study

As a field study employing quantitative methodology and focusing on the intention of Greek school teachers to accept EAIT, the conclusions are neither generalisable nor directly applicable to other educational contexts. Additionally, the reliance on self-reported data introduces potential biases, as participants may overestimate their acceptance and familiarity with EAIT. Furthermore, while the research incorporates various factors influencing acceptance, it does not account for external variables such as school leadership support or parental attitudes towards technology in education. Moreover, the survey has only examined teachers’ tendency to adopt EAIT, and thus does not fully capture the nuances of teachers’ attitudes on EAIT implementation in the classroom or their actual use.

### 4.2. Future Research Studies

On the basis of these limitations, the final results can serve as a foundation for future research to explore a deeper understanding of the complexities surrounding EAIT acceptance. Longitudinal studies may provide insights into how teachers’ perceptions evolve as they gain experience with EAIT. Additionally, expanding the research framework to include external factors could yield a more comprehensive understanding of the potential integration of educational AI.

## 5. Conclusions

This study makes a contribution to the field of AI adoption in education by targeting Greek school teachers. The principal goal is to investigate the interaction of constructs from the TAM, the UTAUT, and additional variables, namely perceived trust, personal innovativeness, facilitating conditions, and pedagogical beliefs, both between them and in relation to teachers’ behavioural intention to accept EAIT in their practice. To the best of our knowledge, this project is the first comprehensive investigation of the causal relationships between the aforementioned factors and teachers’ tendency to use EAIT in the context of school education. The research findings indicate the significant role of perceived usefulness as the most dominant positive and significant predictor of teachers’ behavioural intention to use EAIT. This is followed by personal innovativeness and social influence in promoting EAIT acceptance.

Overall, the findings highlight the need for targeted professional development that addresses the specific concerns and needs of teachers, ensuring they are adequately supported in their transition to using AI tools in their classrooms. An understanding of the results enables the development of effective strategies for the implementation of educational AI, with the aim of improving teachers’ instructional performance and consequently students’ learning experiences. The rigorous methodological approach of PLS-SEM, applied to the empirical findings, provides valuable insights for educational institutions and policymakers to develop targeted interventions that facilitate the effective and responsible incorporation of AI in education. This includes fostering a culture of innovation and trust and providing adequate resources and support, such as the development of professional programmes, which promote student-oriented pedagogical approaches and align with the potential of EAIT.

## Figures and Tables

**Table 1:** Definitions of the acronyms (in alphabetical order).

Variable	Acronym
Constructivist pedagogical beliefs	CPB
Behavioural intention	BI
Facilitating conditions	FC
Perceived ease of use	PEU
Perceived trust	PT
Perceived usefulness	PU
Personal innovativeness	PI
Social influence	SI
Traditional pedagogical beliefs	TPB

**Table 2:** Descriptive analysis of the sample.

Demographic	Group	Frequency	Percentage
Gender	Male	76	22.2%
	Female	265	77.5%
	Prefer not to disclose	1	0.3%
Age	21–30	30	8.8%
	31–40	52	15.2%
	41–50	118	34.5%
	51+	142	41.5%
Academic qualifications	Bachelor	139	40.6%
	Master	171	50%
	PhD	32	9.4%
Teaching experience	<10	83	24.3%
	11–20	101	29.5%
	21–30	111	32.5%
	>31	47	13.7%
PD on AI expertise	Yes	227	66.4%
	No	115	33.6%

**Table 3:** Measurement scales.

Construct	Item No.	Item	Reference
Traditional pedagogical beliefs (TPB)	TPB1	During the lesson, it is important that students are confined to their books and desks.	Choi et al. [24]
	TPB2	The art of teaching is simply the application of the theories of university teachers in practice, without questioning them.	
	TPB3	Teaching is exclusively concerned with the introduction, presentation or explanation of the subject matter.	
	TPB4	The teacher must provide students with accurate and complete knowledge, rather than encourage them to discover it.	
	TPB5	Effective teaching is achieved when the classroom is dominated by the teacher’s lecture.	
Constructivist pedagogical beliefs (CPB)	CPB1	The learning process requires that students have ample opportunities to explore, discuss and express their ideas.	Choi et al. [24]
	CPB2	Every student is unique and deserves an education tailored to their specific needs.	
	CPB3	It is important for the teacher to understand the emotions of the students.	
	CPB4	Effective teachers should encourage students to explore possible answers autonomously.	
	CPB5	In classrooms where effective teaching practices are employed, there is a democratic and free atmosphere that encourages students to think and interact.	
Personal innovativeness (PI)	PI1	I like to experiment with new information technologies (IT).	Strzelecki [42]
	PI2	When I hear about a new IT, I look for ways to experiment with it.	
	PI3	I am often among the first in my social circle to try out a new IT.	
	PI4	Overall, I do not hesitate to try out a new IT.	
Perceived trust (PT)	PT1	I think that EAIT can generate reliable results.	Choi et al. [24]
	PT2	I think that EAIT render fair decisions.	
	PT3	I believe that EAIT are trustworthy.	
	PT4	Overall, I can trust EAIT.	
Perceived usefulness (PU)	PU1	Using EAIT at work would boost my productivity.	Choi et al. [24]
	PU2	Using EAIT would improve my performance at work.	
	PU3	The use of EAIT would enhance my effectiveness in my work.	
	PU4	I find EAIT useful in my work.	
Perceived ease of use (PEU)	PEU1	My interaction with the EAIT is clear and understandable.	Choi et al. [24]
	PEU2	The use of EAIT does not require much mental effort.	
	PEU3	I find that EAIT are easy to use.	
	PEU4	I find that I can easily drive the system to do what I want it to do.	
Social influence (SI)	SI1	The people who are important to me think I should use EAIT.	Strzelecki [42]
	SI2	The people who influence my behaviour think I should use EAIT.	
	SI3	The people whose opinions I value urge me to use EAIT.	
Facilitating conditions (FC)	FC1	I have the necessary resources to use EAIT.	Strzelecki [42]
	FC2	I have the necessary knowledge to use EAIT.	
	FC3	EAIT are compatible with the technologies I use.	
	FC4	I can ask others for help if I have difficulties using EAIT.	
Behavioural intention (BI)	BI1	Assuming I have access, I intend to use EAIT.	Choi et al. [24]
	BI2	If I had access to EAIT, I predict that I would use them.	
	BI3	In the future I plan to use EAIT in my school work.	

**Table 4:** Construct reliability and validity.

	Cronbach’s α	Composite Reliability (rho_a)	Composite Reliability (rho_c)	Average Variance Extracted (AVE)
BI	0.927	0.928	0.954	0.874
CPB	0.840	0.850	0.886	0.610
FC	0.813	0.864	0.879	0.653
PEU	0.806	0.871	0.867	0.626
PI	0.897	0.906	0.928	0.764
PT	0.899	0.904	0.930	0.768
PU	0.943	0.943	0.959	0.854
SI	0.934	0.934	0.958	0.883
TPB	0.763	0.798	0.833	0.502

**Table 5:** Discriminant validity of the measurement model based on the Fornell–Larcker criterion.

	BI	CPB	FC	PEU	PI	PT	PU	SI	TPB
BI	0.935								
CPB	0.352	0.781							
FC	0.464	0.151	0.808						
PEU	0.542	0.177	0.681	0.791					
PI	0.602	0.302	0.530	0.533	0.874				
PT	0.468	0.197	0.338	0.447	0.391	0.876			
PU	0.721	0.291	0.434	0.547	0.459	0.551	0.924		
SI	0.501	0.136	0.406	0.446	0.343	0.454	0.556	0.940	
TPB	−0.263	−0.567	−0.131	−0.144	−0.249	−0.100	−0.164	−0.065	0.708

**Table 6:** Cross-loadings matrix (higher loadings across lines are written in bold).

	BI	CPB	FC	PEU	PI	PU	SI	PT	TPB
BI1	**0.951**	0.337	0.466	0.517	0.552	0.664	0.477	0.413	−0.263
BI2	**0.952**	0.361	0.417	0.517	0.583	0.673	0.474	0.442	−0.271
BI3	**0.901**	0.289	0.419	0.484	0.553	0.683	0.455	0.457	−0.203
CPB1	0.291	**0.825**	0.178	0.193	0.290	0.272	0.135	0.175	−0.513
CPB2	0.288	**0.818**	0.130	0.136	0.220	0.250	0.077	0.156	−0.449
CPB3	0.267	**0.730**	0.027	0.079	0.199	0.197	0.064	0.128	−0.454
CPB4	0.230	**0.754**	0.105	0.086	0.193	0.168	0.098	0.150	−0.391
CPB5	0.292	**0.772**	0.128	0.172	0.258	0.229	0.147	0.156	−0.397
FC1	0.408	0.119	**0.887**	0.585	0.451	0.362	0.341	0.254	−0.129
FC2	0.448	0.131	**0.892**	0.656	0.536	0.428	0.365	0.347	−0.111
FC3	0.374	0.154	**0.856**	0.567	0.433	0.366	0.347	0.264	−0.129
FC4	0.233	0.077	**0.546**	0.337	0.232	0.210	0.252	0.216	−0.033
PEU1	0.538	0.168	0.619	**0.846**	0.555	0.551	0.442	0.496	−0.126
PEU2	0.158	−0.031	0.302	**0.547**	0.154	0.127	0.141	0.080	0.041
PEU3	0.450	0.142	0.566	**0.856**	0.388	0.453	0.344	0.331	−0.124
PEU4	0.433	0.186	0.580	**0.870**	0.448	0.442	0.381	0.348	−0.161
PI1	0.581	0.304	0.451	0.472	**0.901**	0.454	0.362	0.340	−0.261
PI2	0.558	0.320	0.491	0.504	**0.912**	0.445	0.381	0.375	−0.238
PI3	0.435	0.180	0.453	0.428	**0.799**	0.339	0.202	0.290	−0.122
PI4	0.518	0.234	0.460	0.457	**0.879**	0.353	0.229	0.357	−0.236
PU1	0.658	0.280	0.419	0.516	0.436	**0.923**	0.505	0.518	−0.166
PU2	0.647	0.255	0.381	0.480	0.405	**0.944**	0.513	0.469	−0.140
PU3	0.653	0.285	0.364	0.471	0.415	**0.935**	0.472	0.502	−0.140
PU4	0.700	0.255	0.437	0.550	0.436	**0.893**	0.562	0.544	−0.158
SI1	0.471	0.125	0.431	0.461	0.339	0.545	**0.938**	0.449	−0.086
SI2	0.468	0.119	0.362	0.413	0.317	0.517	**0.951**	0.411	−0.036
SI3	0.474	0.138	0.353	0.384	0.311	0.506	**0.930**	0.419	−0.062
T1	0.388	0.204	0.321	0.385	0.360	0.450	0.326	**0.834**	−0.088
T2	0.384	0.138	0.266	0.364	0.310	0.441	0.397	**0.855**	−0.077
T3	0.426	0.207	0.291	0.400	0.349	0.514	0.429	**0.919**	−0.094
T4	0.440	0.141	0.305	0.415	0.351	0.522	0.435	**0.896**	−0.091
TPB1	−0.214	−0.375	−0.132	−0.163	−0.205	−0.180	−0.152	−0.153	**0.761**
TPB2	−0.145	−0.301	−0.015	−0.017	−0.118	−0.038	0.019	0.048	**0.590**
TPB3	−0.190	−0.403	−0.110	−0.113	−0.202	−0.108	0.004	−0.065	**0.725**
TPB4	−0.186	−0.453	−0.026	0.009	−0.112	−0.089	0.044	0.009	**0.669**
TPB5	−0.185	−0.491	−0.113	−0.132	−0.203	−0.103	−0.048	−0.082	**0.780**

**Table 7:** Heterotrait–monotrait ratio (HTMT).

	Heterotrait-Monotrait Ratio (HTMT)
CPB <-> BI	0.396
FC <-> BI	0.526
FC <-> CPB	0.175
PEU <-> BI	0.575
PEU <-> CPB	0.196
PEU <-> FC	0.789
PI <-> BI	0.657
PI <-> CPB	0.337
PI <-> FC	0.605
PI <-> PEU	0.572
PT <-> BI	0.512
PT <-> CPB	0.226
PT <-> FC	0.395
PT <-> PEU	0.464
PT <-> PI	0.434
PU <-> BI	0.769
PU <-> CPB	0.321
PU <-> FC	0.486
PU <-> PEU	0.566
PU <-> PI	0.494
PU <-> PT	0.596
SI <-> BI	0.539
SI <-> CPB	0.150
SI <-> FC	0.468
SI <-> PEU	0.474
SI <-> PI	0.367
SI <-> PT	0.494
SI <-> PU	0.592
TPB <-> BI	0.305
TPB <-> CPB	0.703
TPB <-> FC	0.155
TPB <-> PEU	0.183
TPB <-> PI	0.279
TPB <-> PT	0.124
TPB <-> PU	0.170
TPB <-> SI	0.094

**Table 8:** R2 values of the model’s endogenous variables.

Dependent Variable	R^2^ Coefficient	*t*	*p*
BI	0.635	17.761	0.000
PEU	0.284	6.858	0.000
PT	0.039	1.704	0.088
PU	0.389	8.357	0.000

**Table 9:** Collinearity statistics of model (VIF).

	VIF
CPB -> BI	1.596
CPB -> PEU	1.535
CPB -> PT	1.473
CPB -> PU	1.552
FC -> BI	2.047
PEU -> BI	2.317
PI -> BI	1.687
PI -> PEU	1.111
PI -> PU	1.275
PT -> BI	1.576
PT -> PU	1.194
PU -> BI	2.037
SI -> BI	1.591
TPB -> BI	1.496
TPB -> PEU	1.488
TPB -> PT	1.473
TPB -> PU	1.492

**Table 10:** Direct, indirect, and total effects of the research model.

	Direct	*p*	Total Indirect	*p*	Total	*p*
CPB -> BI	0.073	0.099	0.110	0.001	0.183	0.001
CPB -> PEU	0.017	0.765			0.017	0.765
CPB -> PT	0.207	0.002			0.207	0.002
CPB -> PU	0.144	0.013	0.088	0.006	0.232	0.000
FC -> BI	0.012	0.824			0.012	0.824
PEU -> BI	0.064	0.227			0.064	0.227
PI -> BI	0.276	0.000	0.152	0.000	0.428	0.000
PI -> PEU	0.527	0.000			0.527	0.000
PI -> PU	0.255	0.000			0.255	0.000
PT -> BI	0.007	0.873	0.198	0.000	0.205	0.000
PT -> PU	0.426	0.000			0.426	0.000
PU -> BI	0.465	0.000			0.465	0.000
SI -> BI	0.098	0.035			0.098	0.035
TPB -> BI	−0.059	0.205	0.014	0.649	−0.045	0.366
TPB -> PEU	−0.003	0.965			−0.003	0.965
TPB -> PT	0.017	0.810			0.017	0.810
TPB -> PU	0.024	0.673	0.007	0.811	0.031	0.612

## Data Availability

The data that support the reported results are not publicly available due to privacy and ethical restrictions of the University of Macedonia Ethics Committee’s approval.

## References

[1] Pedro F., Subosa M., Rivas A., Valverde P. Artificial Intelligence in Education: Challenges and Opportunities for Sustainable Development—UNESCO Digital Library. https://unesdoc.unesco.org/ark:/48223/pf0000366994?posInSet=22&queryId=9d8ca6cf-6a26-4f09-9b10-5e339c0e75da.

[2] Haenlein M., Kaplan A. (2019). A Brief History of Artificial Intelligence: On the Past, Present, and Future of Artificial Intelligence. Calif. Manag. Rev..

[3] Chita E.-I., Dumitrescu-Popa S., Motorga B., Panait M. (2023). Artificial Intelligence—Source of Inspiration or a Problem?. Proc. Int. Conf. Bus. Excell..

[4] The Future of Jobs Report 2020. https://www.weforum.org/reports/the-future-of-jobs-report-2020/digest/.

[5] Tuomi I. The Impact of Artificial Intelligence on Learning, Teaching, and Education. https://publications.jrc.ec.europa.eu/repository/handle/JRC113226.

[6] Harari Y.N. (2017). Reboot for the AI Revolution. Nature.

[7] Lampropoulos G., Geroimenko V. (2023). Augmented Reality and Artificial Intelligence in Education: Toward Immersive Intelligent Tutoring Systems. Augmented Reality and Artificial Intelligence: The Fusion of Advanced Technologies.

[8] MinnaLearn and the University of Helsinki A Free Online Introduction to Artificial Intelligence for Non-Experts. https://course.elementsofai.com/.

[9] Baker T., Smith L., Anisa N. Educ-AI-Tion Rebooted? Exploring the Future of Artificial Intelligence in Schools and Colleges. https://www.nesta.org.uk/report/education-rebooted/.

[10] Lampou R. (2023). The Integration of Artificial Intelligence in Education: Opportunities and Challenges. Rev. Artif. Intell. Educ..

[11] Zawacki-Richter O., Marín V.I., Bond M., Gouverneur F. (2019). Systematic Review of Research on Artificial Intelligence Applications in Higher Education—Where Are the Educators?. Int. J. Educ. Technol. High. Educ..

[12] Holmes W., Tuomi I. (2022). State of the Art and Practice in AI in Education. Eur. J. Educ..

[13] Zaman B.U. (2023). Transforming Education through AI, Benefits, Risks, and Ethical Considerations. https://www.techrxiv.org/doi/full/10.36227/techrxiv.24231583.v1.

[14] Rahman M.M., Watanobe Y. (2023). ChatGPT for Education and Research: Opportunities, Threats, and Strategies. Appl. Sci..

[15] Vidal J., Llorens-Largo F., García-Peñalvo F.J. (2024). The New Reality of Education in the Face of Advances in Generative Artificial Intelligence. Rev. Iberoam. De Educ. A Distancia.

[16] Sullivan M., Kelly A., McLaughlan P. (2023). ChatGPT in Higher Education: Considerations for Academic Integrity and Student Learning. J. Appl. Learn. Teach..

[17] Kizilcec R.F. (2023). To Advance AI Use in Education, Focus on Understanding Educators. Int. J. Artif. Intell. Educ..

[18] Nazaretsky T., Cukurova M., Alexandron G. An Instrument for Measuring Teachers’ Trust in AI-Based Educational Technology. Proceedings of the LAK22: 12th International Learning Analytics and Knowledge Conference.

[19] Adiguzel T., Kaya M.H., Cansu F.K. (2023). Revolutionizing Education with AI: Exploring the Transformative Potential of ChatGPT. Cont. Ed. Technol..

[20] Eurydice Organisation of the Education System and of Its Structure. https://eurydice.eacea.ec.europa.eu/national-education-systems/greece/organisation-education-system-and-its-structure.

[21] Foutsitzi S., Caridakis G. (2021). Aspects Affecting the Use of Digital Technologies in Greek Schools. Int. Educ. Stud..

[22] Lazaridou A., Dimou N., Korkakaki N., Nousia E. The Use of New Technologies in Greek Schools: Teachers’ Perspectives Concerning New Technologies and the School Principal’s Role as a Technology Leader. Proceedings of the Edulearn10.

[23] Holmes W., Bialik M., Fadel C. (2023). Artificial Intelligence in Education. Data Ethics: Building Trust: How Digital Technologies Can Serve Humanity.

[24] Choi S., Jang Y., Kim H. (2023). Influence of Pedagogical Beliefs and Perceived Trust on Teachers’ Acceptance of Educational Artificial Intelligence Tools. Int. J. Hum. Comput. Interact..

[25] Chocarro R., Cortiñas M., Marcos-Matás G. (2023). Teachers’ Attitudes towards Chatbots in Education: A Technology Acceptance Model Approach Considering the Effect of Social Language, Bot Proactiveness, and Users’ Characteristics. Educ. Stud..

[26] Ayanwale M.A., Sanusi I.T., Adelana O.P., Aruleba K.D., Oyelere S.S. (2022). Teachers’ Readiness and Intention to Teach Artificial Intelligence in Schools. Comput. Educ. Artif. Intell..

[27] An X., Chai C.S., Li Y., Zhou Y., Shen X., Zheng C., Chen M. (2023). Modeling English Teachers’ Behavioral Intention to Use Artificial Intelligence in Middle Schools. Educ. Inf. Technol..

[28] Emon M.M.H., Hassan F., Nahid M.H., Rattanawiboonsom V. (2023). Predicting Adoption Intention of Artificial Intelligence. AIUB J. Sci. Eng..

[29] Davis F.D. (1989). Perceived Usefulness, Perceived Ease of Use, and User Acceptance of Information Technology. MIS Q..

[30] Venkatesh V., Morris M.G., Davis G.B., Davis F.D. (2003). User Acceptance of Information Technology: Toward a Unified View. MIS Q..

[31] Wang Y., Liu C., Tu Y.-F. (2021). Factors Affecting the Adoption of AI-Based Applications in Higher Education: An Analysis of Teachers Perspectives Using Structural Equation Modeling. Educ. Technol. Soc..

[32] Vincent-Lancrin S., van der Vlies R. (2020). Trustworthy Artificial Intelligence (AI). Education: Promises and Challenges.

[33] Qin F., Li K., Yan J. (2020). Understanding User Trust in Artificial Intelligence-Based Educational Systems: Evidence from China. Br. J. Educ. Technol..

[34] Tondeur J., van Braak J., Ertmer P.A., Ottenbreit-Leftwich A. (2017). Understanding the Relationship between Teachers’ Pedagogical Beliefs and Technology Use in Education: A Systematic Review of Qualitative Evidence. Educ. Tech. Res. Dev..

[35] Burke P.F., Schuck S., Aubusson P., Kearney M., Frischknecht B. (2018). Exploring Teacher Pedagogy, Stages of Concern and Accessibility as Determinants of Technology Adoption. Technol. Pedagog. Educ..

[36] Oyunge T.O. (2021). Exploring secondary school teachers’ pedagogical beliefs and the integration of ICT in the context of a developing country: A technology acceptance model perspective. Eur. J. Educ. Stud..

[37] Liu H., Lin C.-H., Zhang D. (2017). Pedagogical Beliefs and Attitudes toward Information and Communication Technology: A Survey of Teachers of English as a Foreign Language in China. Comput. Assist. Lang. Learn..

[38] Gurer M.D., Akkaya R. (2022). The Influence of Pedagogical Beliefs on Technology Acceptance: A Structural Equation Modeling Study of Pre-Service Mathematics Teachers. J. Math. Teacher Educ..

[39] Mazman Akar S.G. (2019). Does It Matter Being Innovative: Teachers’ Technology Acceptance. Educ. Inf. Technol..

[40] Agarwal R., Prasad J. (1998). A Conceptual and Operational Definition of Personal Innovativeness in the Domain of Information Technology. Inf. Syst. Res..

[41] Şahin F., Dursun Ö. (2022). Does Innovativeness Matter in Technology Adoption? Addressing Pre-Service Teachers’ Intention to Use ITs. JETOL.

[42] Strzelecki A. (2023). To Use or Not to Use ChatGPT in Higher Education? A Study of Students’ Acceptance and Use of Technology. Interact. Learn. Environ..

[43] Twum K.K., Ofori D., Keney G., Korang-Yeboah B. (2021). Using the UTAUT, Personal Innovativeness and Perceived Financial Cost to Examine Student’s Intention to Use E-Learning. J. Sci. Technol. Policy Manag..

[44] Aldholay A., Isaac O., Jalal A.N., Anor F.A., Mutahar A.M., Al-Emran M., Al-Sharafi M.A., Al-Kabi M.N., Shaalan K. (2022). Factors That Accelerate the Rise of Acceptance of Big Data Platforms for Academic Teaching: Personal Innovativeness as Moderating Variable. Proceedings of the International Conference on Emerging Technologies and Intelligent Systems 2021.

[45] Wu W., Zhang B., Li S., Liu H. (2022). Exploring Factors of the Willingness to Accept AI-Assisted Learning Environments: An Empirical Investigation Based on the UTAUT Model and Perceived Risk Theory. Front. Psychol..

[46] Digital Transformation Bible 2020–2025. https://digitalstrategy.gov.gr/en/sector/digin_ai.

[47] Wang G., Shin C. (2022). Influencing Factors of Usage Intention of Metaverse Education Application Platform: Empirical Evidence Based on PPM and TAM Models. Sustainability.

[48] Sánchez-Prieto J.C., Cruz-Benito J., Therón R., García-Peñalvo F. (2020). Assessed by Machines: Development of a TAM-Based Tool to Measure AI-Based Assessment Acceptance Among Students. IJIMAI.

[49] Prasetyo Y.T., Ong A.K.S., Concepcion G.K.F., Navata F.M.B., Robles R.A.V., Tomagos I.J.T., Young M.N., Diaz J.F.T., Nadlifatin R., Redi A.A.N.P. (2021). Determining Factors Affecting Acceptance of E-Learning Platforms during the COVID-19 Pandemic: Integrating Extended Technology Acceptance Model and DeLone & McLean IS Success Model. Sustainability.

[50] Williams M.D., Rana N.P., Dwivedi Y.K. (2015). The Unified Theory of Acceptance and Use of Technology (UTAUT): A Literature Review. J. Enterp. Inf. Manag..

[51] Almaiah M.A., Alamri M.M., Al-Rahmi W. (2019). Applying the UTAUT Model to Explain the Students’ Acceptance of Mobile Learning System in Higher Education. IEEE Access.

[52] Chao C.-M. (2019). Factors Determining the Behavioral Intention to Use Mobile Learning: An Application and Extension of the UTAUT Model. Front. Psychol..

[53] Sabri M.F.M., Baba N., Sulaiman W.A.N.W. (2023). Investigating Hospitality Student’s Acceptance in Online Learning Platform: Utilising UTAUT Model. Int. J. Acad. Res. Bus. Soc. Sci..

[54] Taddeo M. (2009). Defining Trust and E-Trust: From Old Theories to New Problems. IJTHI.

[55] Schmidt P., Biessmann F., Teubner T. (2020). Transparency and Trust in Artificial Intelligence Systems. J. Decis. Syst..

[56] König J. (2012). Teachers’ Pedagogical Beliefs. Definition and Operationalisation, Connections to Knowledge and Performance, Development and Change.

[57] Shi K. (2022). Contrasting Behaviorist and Constructivist Perspectives on Learning for Students with Emotional and Behavioral Disorders.

[58] Lu J., Yao J.E., Yu C.-S. (2005). Personal Innovativeness, Social Influences and Adoption of Wireless Internet Services via Mobile Technology. J. Strateg. Inf. Syst..

[59] Ringle C.M., Wende S., Becker J.-M. SmartPLS 4. Bönningstedt: SmartPLS 2024. https://www.smartpls.com/.

[60] Civelek M. (2018). Essentials of Structural Equation Modeling.

[61] Hair J.F., Ringle C.M., Sarstedt M. (2013). Partial Least Squares Structural Equation Modeling: Rigorous Applications, Better Results and Higher Acceptance. Long Range Plan..

[62] Hair J.F., Hult G.T.M., Ringle C.M., Sarstedt M., Danks N.P., Ray S. (2021). Partial Least Squares Structural Equation Modeling (PLS-SEM) Using R: A Workbook.

[63] Hair J., Alamer A. (2022). Partial Least Squares Structural Equation Modeling (PLS-SEM) in Second Language and Education Research: Guidelines Using an Applied Example. Res. Methods Appl. Linguist..

